# Germ cell tumor and Takotsubo Cardiomyopathy: A treatment dilemma

**DOI:** 10.12669/pjms.344.15293

**Published:** 2018

**Authors:** Abdul Hannan, Muhammad Faisal Khalid, Samia Yasmeen

**Affiliations:** 1 Dr. Abdul Hannan, MBBS, MD, FCPS, FCPS, Board Eligible - American Board of Internal Medicine, FACP. Chief Resident – Internal Medicine, **Past Affiliation:** Consultant Medical Oncologist, Shaukat Khanum Memorial Cancer Hospital and Research Center, Lahore, Pakistan. East Tennessee State University / James H. Quillen College of Medicine, Johnson City, Tennessee. USA. 37601; 2 Dr. Muhammad Faisal Khalid, MBBS, MD. Resident Internal Medicine, East Tennessee State University / James H. Quillen College of Medicine, Johnson City, Tennessee. USA. 37601; 3 Dr. Samia Yasmeen, MBBS, FCPS, FCPS. Consultant Medical Oncologist, Shaukat Khanum Memorial Cancer Hospital and Research Center, Lahore, Pakistan

**Keywords:** Carboplatin, Cardiomyopathy, Cisplatin, Germ cell tumor, Takatsubo

## Abstract

Germ cell tumors (GCT) are uncommon malignancies in adult males and comprise less than 1% of male cancers. Due to highly curative nature and productive life years gained after treatment; reduction of chemotherapy related toxicities becomes vital. Cisplatin is the backbone of GCT chemotherapy, & is related to myocardial injury, thromboembolism & vasculitis. Though it should not be replaced with Carboplatin, however in certain circumstances, its use maybe unsafe; especially in cases when patient have prior myocardial infarction. We report a case of Takotsubo cardiomyopathy (TCM)secondary to GCT diagnosis in a young male. This patient presented with symptoms of myocardial infarction however, coronary angiography was normal and a diagnosis of TCM was made. Though, it is rare but a unique challenge, as whether Cisplatin use would be safe in this particular scenario? On one hand patient had stress related myocardial injury while he was also at risk of further Cisplatin induced complications. There are no clear cut guidelines, so after informed consent his treatment regimen was modified to EC (Etoposide/Carboplatin) instead of EP (Etoposide/Cisplatin). Patient has completed 4.6 years of follow-up without any evidence of relapse. We suggest informed decisions and to weigh the pros and cons of using an inferior regimen, in order to achieve same long term prognosis while preventing any acute complications, in younger patients with curable cancers.

## INTRODUCTION

Germ cell tumors (GCT) are a classic example of cure among solid malignancies. Cure has been achieved in almost 80% of cases with 5-year survival rates of more than 95% in developed countries.^1^ Reducing the treatment related toxicities becomes more important with high cure rates. Off note, Cisplatin is an integral component of GCT chemotherapy & it can’t be replaced with any other agents like carboplatin, as direct comparisons have consistently showed inferior outcomes.^2^,^3^ However, sometimes there are unique treatment challenges in cases where use of Cisplatin may be related to poor outcomes or complications like vasculitis. Here we report a patient with newly diagnosed GCT, who developed Takatsubo Cardiomyopathy (TCM) due to stress of cancer diagnosis and had a compelling indication to change the treatment regimen. Though not the standard of cure with very limited data available for use of Carboplatin, keeping the balance in side effects and long term prognosis is important in treatment of highly curable malignancies, especially in young patients, which is highlighted in this case report.

## CASE REPORT

**Fig.1:**
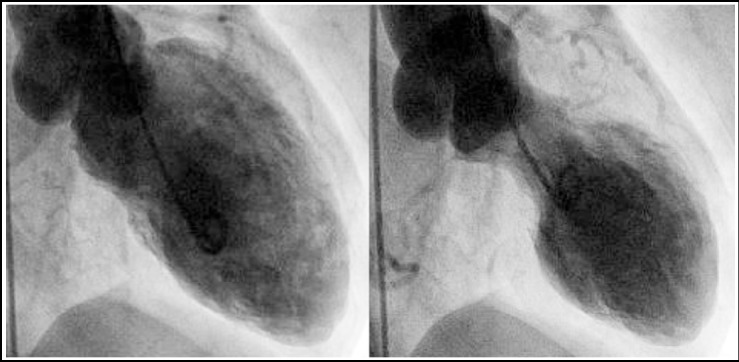
Cardiac Catheterization images showing normal diastolic left ventricular cavity (Left) and anterior and apical ballooning during systole.

A 32 years old male presented with painless and gradually enlarging right testicular swelling of 3 months duration. Right inguinal orchiectomy was performed and 3 weeks post Op B-hCG was 2900IU, AFP was 890 ng/ml while LDH was 560mg/ml. Histopathology showed yolk sac tumor (65%), immature teratoma (20%) and seminoma (15%). Baseline CT scan showed a 1.8cm right para-aortic lymph node with no other metastatic disease. Patient was staged as Stage IIA-S1, Good risk Mixed Germ Cell Tumor (MGCT) depending upon the markers & CT findings. Chemotherapy was planned within 3 weeks of surgery; however patient presented after 2^nd^ week of orchiectomy to ER with severe chest pain. At presentation, cardiac enzymes were elevated and his Trop-I was 6.2 (normal < 0.4), while EKG showed 1.8mm ST segment elevation in leads V1-V3. Cardiology team was consulted immediately & echocardiogram was performed which showed apical hypokinesis with EF of 49%. His cardiac catheterization was reported to be normal with normal coronaries and without any flow restriction (Fig.1). Depending upon the presentation, EKG changes, elevated Trop-I with absence of any flow restriction and normal coronary angiogram, TCM diagnosis was established, based upon Mayo clinic diagnostic criteria (Table-I).^4^ Patient was started on angiotensin converting enzyme inhibitors and beta-blockers, and his chemotherapy was delayed by 3 weeks.

**Table-I:** Mayo clinic Diagnostic Criteria for Takotsubo cardiomyopathy.^4^

1.	Suspected acute MI based on symptoms, ECG changes, &/or elevation of cardiac enzymes
2.	Transient LV wall motion abnormalities extending beyond a single epicardial vascular distribution.
3.	Absence of potentially culpritcoronary stenosis by angiography (≤ 50% luminal narrowing of the epicardial arteries and no angiographic evidence of acute plaque rupture.
4.	No clinical indicator of myocarditis or pheochromocytoma.

Later his chemotherapy regimen was modified to EC x 4 (Etoposide 100 mg/m^2^IV Day 1-5 / Carboplatin AUC-5 IV Day 1 only) instead of EP (Etoposide / Cisplatin). Though there is scarce data regarding the use of Carboplatin in MGCT and Cisplatin has been shown to be superior to Carboplatin, however due to a risk of Cisplatin induced vasculitis or acute thrombosis, the treatment regimen was modified with informed consent. Patient was treated with 4 cycles of EC and after 2^nd^ cycle his markers normalized. At this time decision was reviewed to cut down his chemotherapy to only 3 cycles of EC but it was considered that he is already getting sub-optimal regimen, so he was continued with complete 4 cycles. He tolerated his treatment well, achieved complete biochemical and radiological response and placed on surveillance. Patient has completed 4.6 years of follow-up according to National Comprehensive Cancer Network guidelines and remains well without any evidence of relapse. After 6 months of his treatment with ACEi and BBs, both were also stopped as he had normal EF (62%) on echocardiogram.

## DISCUSSION

Takotsubo cardiomyopathy (TCM), also known as “stress cardiomyopathy” or “broken heart syndrome” was first described in Japan in 1990 by Sato et al. with increased recognition throughout the world thereafter.^5^ TCM is a transient reversible left ventricular systolic dysfunction associated with stress, mimicking ST segment elevation myocardial infarctionon electrocardiography.^5^ It has been reported in 1-2% of patients who present with troponin positive acute coronary syndrome.^6^

Studies have reported a higher incidence of TCM among cancer patients (10%), with different types of cancers, chemotherapeutic agents, tyrosine kinase inhibitors & monoclonal antibodies.^7^ The relationship between Takotsubo cardiomyopathy, malignancy diagnosis & chemotherapy remains mysterious. Adrenergic excess, coronary artery vasospasm & microvascular dysfunction have been reported as possible mechanisms.^8^ Studies have suggested that cancer can lower the threshold for stress stimuli and may aggravate cardiac adrenoreceptor sensitivity predisposing patients to develop TCM. Among all the proposed mechanisms, adrenergic surge due to stress is most plausible as diagnosis of cancer is a significant stressor, leading to coronary micro-vascular spasm resulting in myocardial infarction.^9^ However, why it only involves apical or anterior segment, is unknown. The other postulated explanation is as part of cancer-related paraneoplastic syndrome, which remains to be further elucidated.

Our case is unique as it was not related to chemotherapy, rather TCM happened due to stress of cancer diagnosis before chemotherapy treatment& led us to modify the treatment regimen. The standard of care for Stage IIA - Good risk MGCT is either four cycles of EP regimen (Etoposide 100mg/m^2^, Cisplatin 20 mg/m^2^) or three cycles of BEP regimen (Bleomycin 30 units every week, Etoposide 100mg/m^2^, Cisplatin 20 mg/m^2^), every three weeks.^10^ We usually use EP regimen to avoid Bleomycin pulmonary toxicity as an institutional practice, moreover in our patient there was baseline dyspnea secondary to his recent TCM, which precluded Bleomycin use. Though Cisplatin remains the cornerstone of GCT chemotherapy and a standard of care, however it is also related to some other rare side effects like vasculitis, cardiomyopathy and vascular thrombosis. Various different mechanisms has been mentioned for cisplatin induced cardiomyopathy and thrombo-embolism, some of which are endovascular injury with intimal fibrosis, decreased activation of protein C, hypomagnesaemia and myocardial fiber apoptosis.^11^ Due to potential risk of further cardiac complications, it was a treatment challenge as to whether we should use Cisplatin or not, in a patient with a new cardiac event as TCM.

Bajorin DF et al. randomized 270 patients with Good risk GCT to receive either four cycles of EP or EC and showed that the relapse rates were significantly higher (24%) in EC arm, as compared to EP arm (13%).^2^ They also reported inferior event free and relapse free survival for EC arm, however no difference in overall survival was noted. Similarly, Horwich A et al. reported inferior outcomes when they randomized 598 patients with good risk MGCT to either receive BEP or BEC, replacing Cisplatin for Carboplatin.^3^ They concluded that combination chemotherapy with Carboplatin containing regimens is inferior to Cisplatin regimens, and reported 3 year survival rates of 90% as compared to 97%. Though there are no reports or robust data for the use Carboplatinin MGCT patients at increased risk of cardiac complications, however modification of treatment regimen may be needed in special scenarios like the case we report. To the best of our knowledge, this is the first documented report of TCM with significant implication on his treatment requiring chemotherapeutic regimen change.

This case highlights the importance of considering alternate chemotherapeutic agents in patients who are at risk of future cardiovascular events or who have prior myocardial injury. Though Cisplatin containing regimens remain the standard of care in MGCT, Carboplatin may be used and a fair option in special scenarios likes the reported case. We recommend informed discussion before substituting Cisplatin with Carboplatin, especially in younger patients with curable malignancies, in whom careful balance between risks of immediate cardiac side effects with Cisplatin versus slightly compromised long term survival with Carboplatinis of paramount importance.

### Authors Contribution

**AH and SY** treated and followed the patient as primary consultants, conceived the idea, did manuscript writing and editing from chemotherapy point of view.

**MFK** wrote the manuscript especially the cardiology part.

**AH and SY** did final approval of manuscript.
